# Assessing the potential efficacy of 830-nanometer low-level laser therapy in cats: Extraoral applications

**DOI:** 10.14202/vetworld.2024.1124-1129

**Published:** 2024-05-17

**Authors:** Phanthit Kamlangchai, Naruepon Kampa, Thanikul Srithunyarat, Suvaluk Seesupa, Somphong Hoisang, Duangdaun Kaenkangploo, Preenun Jitasombuti, Chalermkwan Nonthakotr, Nitaya Boonbal, Supranee Jitpean

**Affiliations:** 1 Veterinary Teaching Hospital, Faculty of Veterinary Medicine, Khon Kaen University, Khon Kaen, 40002, Thailand; 2 Division of Surgery, Faculty of Veterinary Medicine, Khon Kaen University, Khon Kaen, 40002, Thailand

**Keywords:** chronic gingivitis, near-infrared light, penetration, photobiomodulation, transmission

## Abstract

**Background and Aim:**

Low-level laser therapy (LLLT) has shown benefits as an alternative treatment of feline chronic gingivostomatitis by reducing pain and inflammation within the oral cavity. Extraoral application technique in cats provides more comfort compared to intraoral application. However, the efficacy of LLLT through buccal tissue is still controversial. This study aimed to investigate the penetration efficacy of LLLT using 830 nm continuous waves with various settings and different application techniques.

**Materials and Methods:**

Twenty-four healthy cats were included in this study. The wavelength of LLLT was 830 nm with an output power of 200 mW through extraoral application, using fluences of 2 and 6 J/cm^2^ in continuous-wave mode. This study compared different distances (contact and non-contact) and three different transmission media (absent media, alcohol, and normal saline solution). Measurement of the laser power within the oral cavity is represented as the mean output power (MOP).

**Results:**

Penetration efficacy was detectable for all fluences, distances, and transmission media, with an average buccal thickness of 2.68 mm. MOP did not differ between fluences of 2 and 6 J/cm^2^ (p = 0.19). In the absence of media, MOP was significantly higher compared with alcohol (p < 0.05) but was not significantly different from normal saline solution (p = 0.26).

**Conclusion:**

Extraoral application of LLLT demonstrated penetration efficacy through the buccal tissue with both contact and non-contact skin (<10 mm). This is a potential alternative treatment for oral diseases in clinical practice. However, there is a need for further study on the efficacy of treatment in clinical practice.

## Introduction

Feline chronic gingivostomatitis (FCGS) is a severe inflammation of the oral mucosa in cats, categorized into two clinical phenotypes: Ulcerative and proliferative, with some patients potentially exhibiting both [1, 2]. Recent literature suggests that FCGS may manifest as an immune-mediated condition associated with feline calicivirus infection [2]. These symptoms lead to severe pain, anorexia, and decreased quality of life [1–4]. The prevalence rate of FCGS ranges from 0.7% to 26.6% [2, 4–7]. At present, FCGS treatment aims to alleviate symptoms and oral inflammation. Dental extraction, pain management, and medical treatment, such as corticosteroids, interferons, and cyclosporine, are widely used to manage these conditions [1–3, 8]. Dental extractions, including full mouth extraction (FME) or partial mouth extraction, are performed according to individual criteria [1, 2]. In a retrospective study involving 95 cats with FCGS treated with tooth extraction, more than two-thirds of affected cats achieved significant improvement or complete resolution [9]. In addition, 68.8% of cats showed a significant improvement or a complete resolution and required extended medical treatment. In another study involving 56 cats with FCGS treated with surgical tooth extractions, it was found that 51.8% achieved clinical cure or showed significant improvement within 38 days [10]. Although FME is often recommended, approximately 20% of cats may not improve and may require additional medical therapy [9]. Corticosteroids are often used to treat FCGS to control inflammation, which has shown clinical improvement in the short-term [1, 3, 8]. In a randomized, double-blind, comparative study between prednisolone and interferon in cats, clinical remission was achieved in 7.7% and 10% of patients, respectively, with no significant difference between the two groups [8]. In addition, the potential adverse effects of corticosteroids during long-term use should be carefully considered. Cyclosporine is an immunosuppressive therapy that has shown efficacy in achieving clinical remission in 45.5%–50% of cases after receiving cyclosporine for 3–6 months [11, 12]. However, cyclosporine should be used cautiously for opportunistic infections [13, 14]. Therefore, adjunctive therapeutic options are required in the management of FCGS.

Low-level laser therapy (LLLT) or photobiomodulation stimulates the biostimulation process by applying photons to living cells and subsequently activating the mitochondrial cytochrome c complex [15–17]. This reaction enhances a biological mechanism that improves metabolism within the cell. This process promotes wound healing, reduces pain, and alleviates target tissue inflammation [17–20]. A minimum laser energy of 0.1 mW/cm^2^ is required to induce biostimulation at the cellular level [21]. In LLLT, this effect is achieved using wavelengths between 600 and 1000 nm [22, 23]. LLLT has been clinically utilized as an alternative therapy for various oral conditions, including oral lichen planus, recurrent aphthous stomatitis, and periodontal inflammation [24–28]. The efficacy of LLLT in reducing inflammation, relieving pain, and promoting ulcer healing is considered a reliable alternative to topical steroids [26, 27]. In feline oral cavity, LLLT application may involve an intraoral application method, where laser energy is emitted directly toward the oral cavity by gently manipulating the lips. However, cats suffering from severe pain or inflammation may resist treatment or may become harmful to veterinarians. Therefore, these cats must be sedated before LLLT application [16]. LLLT is recommended to apply the laser outside the oral cavity by contact with the cheek bulge. In cats, this procedure minimizes the need for sedation, restraint, and stress [3, 16]. LLLT can provide maximum therapeutic efficacy by effectively delivering light waves, reaching target tissue, and efficiently absorbing light. The efficacy of this process is determined by wavelength [16, 29, 30]. In general, longer wavelengths are capable of penetrating deeper. However, the transmission of light through material is reduced by scattering and absorption through chromophores, such as water, pigments, tissue proteins, hemoglobin, and melanin [31, 32]. The wavelength range of 760–850 nm has the best penetrating efficacy [33], whereas the wavelength of 830 nm, which is considered near-infrared light, has remarkable penetration efficacy in cadaveric models through the human cheek [30] and various biological tissue samples [29, 34].

This study aimed to evaluate the potential penetration efficacy of the extraoral application method in healthy cats using LLLT of near-infrared light at a wavelength of 830 nm, output power of 200 mW, continuous wave, with various settings (fluence of 2 and 6 J/cm^2^, contact and non-contact), and different media applied to the cheek bulge. These results provide evidence of the efficacy of LLLT through feline buccal tissue, which can be applied in clinical practice.

## Materials and Methods

### Ethical approval and Informed consent

The Institutional Animals Care and Use Committee of Khon Kaen University (IACUC-KKU-79/65) approved this study. The research protocol was informed before the study, and the owners provided written consent.

### Study period and location

This research study was conducted from September 2022 to April 2023. All procedures were performed at Veterinary Teaching Hospital (VTH), Khon Kaen University (KKU), Thailand.

### Animals

Twenty-four healthy cats (four females and 20 males) owned by clients were included in this study. The age and weight of the cat ranged from 1 to 6 years and from 2 to 6 kg, respectively. All cats underwent physical examination, hematology, and clinical biochemistry. These cats were initially classified as healthy cats and later underwent elective neutering at the VTH, KKU from September 2022 to April 2023.

### Experimental protocols

**Figure-1:**
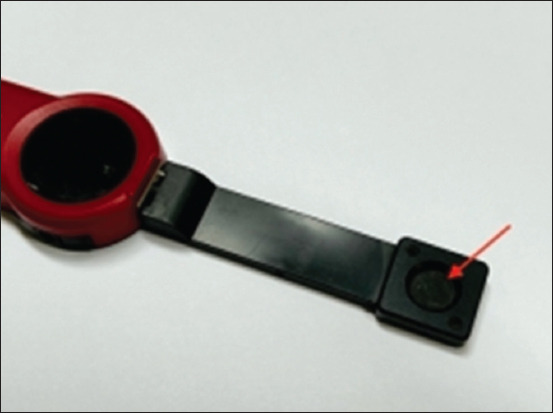
The PM160T wireless thermal power meter is equipped with an ultra-slim sensor that has a diameter of 10 mm (arrow).

**Figure-2:**
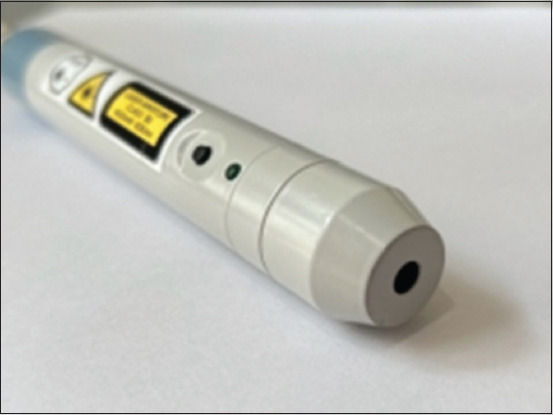
Infrared laser probes with an 830 nm wavelength incorporate green navigation for precise point detection and utilize a small aperture to collimate the beams.

LLLT continuous waveform with a wavelength of 830 nm, an output power of 200 mW, an area of 1 cm^2^, and fluences of 2 and 6 J/cm^2^ were applied extraorally in both groups. A thermal sensor power meter (PM160T, Thorlabs®, USA) placed in contact with the tissue inside the buccal pouch was used to measure the power. An infrared laser probe (Class 3B laser: BTL-5000 series, BTL Industries Ltd., UK) was positioned externally on the left buccal pouch perpendicular to the power meter. The laser power is represented as the mean output power (MOP) (Figures-1 and 2).

After surgical neutering, the cats were kept under anesthesia and maintained in pure oxygen with 1–2% isoflurane. All cats were positioned in the right lateral recumbency. A digital Vernier caliper (PG5010, Enast Awite, China) was used to measure the thickness of the left cheek bulge. MOP measurements were performed by placing a laser probe directly on the power meter and 10 mm above it to serve as the basal MOP (Table-1). Different probe distances and transmission media through the feline buccal pouch were evaluated in all cats. In the study groups, several media were placed on the outer side of the buccal pouch, categorized as (1) absent media, (2) 70% ethyl alcohol solution (ALC), and (3) normal saline solution (NSS). This was performed at fluences of 2 and 6 J/cm^2^ and different distances between the buccal pouch and the probe, at 0 (contact) and 10 mm (non-contact).

**Table 1:** The basal mean output power (air) between fluences.

Fluence	Probe-sensor distance	MOP (mW)
2 J/cm^2^	Contact	125.66 ± 5.64
	10 mm	122.39 ± 5.02
6 J/cm^2^	Contact	133.59 ± 4.97
	10 mm	127.22 ± 3.63

Data is represented as mean ± standard deviation. MOP=Mean output power

### Statistical analysis

This research data were represented as a descriptive analysis (mean and standard deviation) of MOP penetration, which was categorized by fluence (2 and 6 J/cm^2^), buccal probe distances (contact and non-contact), and media (absent media, ALC, and NSS). The Shapiro–Wilk test was used to explore the normal distribution of MOP data for the assumption of normality. A three-way factorial analysis of variance (ANOVA) was used to test the main effects of the fluences, distances of buccal probe, and media on MOP. One-way ANOVA with Bonferroni adjustment was separately analyzed for each group of fluences, distance buccal probe, and media if the interaction effects were significant. Data were analyzed using STATA statistical software version 14.1 (StataCorp LP, USA). Statistical significance was set at p < 0.05.

## Results

The mean age of the cats was 1 ± 0.49 years (range, 1–3 years) and the mean body weight was 3.9 ± 1.0 kg (range, 2–5.7 kg). The average buccal thickness was 2.68 ± 0.67 mm (range, 1.27–3.6 mm). However, no statistically significant difference was observed in MOP based on buccal thickness (p = 0.26).

The overall penetration efficacy based on MOP did not significantly differ between fluences of 2 and 6 J/cm^2^ (p = 0.19).

The contact application method at a fluence of 2 J/cm^2^ resulted in a significantly higher MOP compared to the non-contact method (p < 0.05). However, no significant difference was observed between the buccal-probe distances for a fluence of 6 J/cm^2^ (p = 0.11).

For fluences of 2 and 6 J/cm^2^, MOP was significantly superior in the absence of media compared with ALC (p < 0.05), but no difference was observed when compared to NSS. On the other hand, there was no significant difference in MOP among all transmission media in the non-contact method (Table-2).

**Table 2:** Mean output power of each fluence, distance, and transmission media.

Fluence	MOP (mW) of fluence	Buccal-Probe Distance	MOP (mW) of distance	Transmission Media	MOP (mW) of media
2 J/cm^2^	5.48±3.89	Contact	6.37 ± 4.87^a^	No	7.37 ± 4.02^a^
				ALC	5.60 ± 5.32^b^
				NSS	6.14 ± 5.19^ab^
		10 mm	4.58 ± 2.27^b^	No	4.33 ± 1.74
				ALC	4.74 ± 2.80
				NSS	4.69 ± 2.21
6 J/cm^2^	5.84±3.46	Contact	6.42 ± 3.88	No	8.13 ± 3.90^a^
				ALC	4.67 ± 2.47^b^
				NSS	6.48 ± 4.34^ab^
		10 mm	5.25 ± 2.89	No	4.99 ± 3.21
				ALC	4.96 ± 2.64
				NSS	5.80 ± 2.83

Data are represented as mean ± standard deviation. a, b the different superscript lower-case letters denote significant differences between each method in the same column (p < 0.05). MOP=Mean output power, No=Absent of media, ALC=70% ethyl alcohol solution, NSS=Normal saline solution

## Discussion

This study demonstrated that a wavelength of 830 nm efficiently penetrated the living tissue in cats, reaching a maximum depth of 3.6 mm (fluence of 2 and 6 J/cm^2^), which was the thickest buccal mucosa in this study. In a previous study in dogs, a wavelength of 830 nm achieved a tissue penetration depth of 14 mm. In addition, laser penetration cannot be evaluated when the probe is positioned more than 50 mm from the skin [34, 35]. In another study, 10 cats underwent LLLT for the treatment of FCGS using wavelengths of 808 and 905 nm. Histopathological alterations have been reported within the oral cavity of cats [3]. These findings support the efficacy of laser transmission within the oral cavity of felines. However, the efficacy of LLLT through the cheek bulge in cats remains a topic of debate.

This study suggests that there was no significant difference in MOP when an 830 nm wavelength at fluences of 2 and 6 J/cm^2^ was applied. However, a fluence of 2 J/cm^2^ was observed to significantly increase the MOP compared with the non-contact method. Our findings are consistent with a previous study by Kampa *et al*. [34], where a fluence of 2 J/cm^2^ using the contact skin method significantly increased MOP compared to the non-contact technique at a probe-skin distance of 10 mm. In addition, the penetration efficacy of MOP has been shown to be enhanced using the contact method with a pressure compression probe on the tissue [30, 36, 37]. It should be noted that cats suffering from FCGS may experience stress and pain due to compression from the probe during extraoral application.

In our study, there was no significant difference in the penetration efficacy of LLLT through the oral cavity in cats using a fluence of 6 J/cm^2^, with either the probe applied to the cheek bulge gently or even with a non-contact method at a 10-mm distance. Therefore, LLLT with extraoral application, whether in contact or at a distance within 10 mm, may be used as an alternative treatment for oral diseases in cats. Therefore, further studies in clinical practice in cats with oral diseases are required.

Moreover, a fluence of 6 J/cm^2^ had a slightly higher MOP than a fluence of 2 J/cm^2^. This is in contrast to a previous study by Kwon *et al*. [37] that demonstrated an increase in fluence decreases transmittance. However, its effect is minimal and negligible and has less clinical impact [35]. In addition, an increase in fluence or dose does not affect the absorption of light into the tissue. Tissue-optical properties also remain unaffected by prolonged LLLT emission [35]. In addition, using a fluence of 2 J/cm^2^ takes less time than using a fluence of 6 J/cm^2^ for 10 and 30 s, respectively. Hence, a fluence of 2 J/cm^2^ can be considered desirable, offering benefits such as less irradiation time, reduced need for restraint, and effective transmittance of LLLT in the oral cavity in cats.

To enhance penetration efficacy, the use of transmission media on the treatment surface has been considered to increase the MOP. However, our study demonstrated that MOP measured without the application of media was significantly greater than that measured when ALC was used, whereas it did not differ when compared with NSS. Although a previous study by Ryan and Smith [38] recommended clipping hair alone or supplemental cleansing with an alcohol solution before treatment, our findings confirm that application without hair clipping and using NSS can be suitable in clinical practice, potentially satisfying pet owners. In addition, direct contact of the laser probe with extraoral application requires minimal restraint, making it useful as an alternative treatment method and cat-friendly approach.

### Limitations

Limitations of this study include the potential increase in temperature caused by the conversion of photon energy into kinetic energy through the vibration of molecules, which may generate a small amount of heat leading to discomfort, anxiety, or suffering in addition to FCGS. In our experiment, the cats did not feel uncomfortable after recovering from anesthesia and treatment. In this study, LLLT was applied to the healthy tissue of the oral cavity of cats. Cats with FCGS develop extensive inflammation in the oral mucosa, which increases the thickening of buccal tissue. Therefore, this could potentially affect the penetration efficacy of LLLT through extraoral application. Extraoral application of LLLT in FCGS cats may cause pain, discomfort, or increased sensitivity when the laser probe contacts the buccal area. Therefore, further clinical evaluation of FCGS cases treated with LLLT through extraoral applications is warranted.

## Conclusion

This study demonstrated that LLLT with a wavelength of 830 nm and extraoral application effectively penetrated the buccal tissue of cats. Specifically, using a wavelength of 830 nm in continuous wave mode with an output power of 200 mW and a fluence of 2 and 6 J/cm^2^ at a distance within 10 mm without media was found to be recommended and beneficial for LLLT transmission to the oral mucosa using the extraoral application technique. Further studies are warranted to explore the practical advantages of this technique in clinical scenarios and conditions relevant to feline oral health.

## Authors’ Contributions

SJ, NK, TS, SH, and PK: Designed the study and revised the manuscript. DK, PJ, CN, and NB: Provided input on the study design. PK: Conducted the study, data acquisition, and data collection. SS: Performed the statistical analysis. PK: Prepared the manuscript. All authors have read, reviewed, and approved the final manuscript.
